# Development of a novel ssDNA aptamer targeting neutrophil gelatinase-associated lipocalin and its application in clinical trials

**DOI:** 10.1186/s12967-019-1955-7

**Published:** 2019-06-18

**Authors:** Xiaoqian Hong, Huihui Yan, Fuan Xie, Kaiyu Wang, Qiang Wang, Huijuan Huang, Kunrong Yang, Suhong Huang, Tingting Zhao, Junkai Wang, Yunyun Chen, Kuancan Liu, Xiaopeng Lan

**Affiliations:** 1 Institute for Laboratory Medicine, 900 Hospital of the Joint Logistics Team, Navy Medical University (Second Military Medical University) or Dongfang Hospital, Fuzhou, 350025 Fujian China; 2 Department of Laboratory Medicine, Xiang’an Hospital of Xiamen University, School of Medicine, Xiamen University, Xiamen, 361102 Fujian China; 3 School of Medicine, Xiamen University, Xiamen, 361102 Fujian China; 4 Department of Gynaecology and Obstetrics, 900 Hospital of the Joint Logistics Team or Dongfang Hospital, Fuzhou, 350025 Fujian China; 5 Department of Nephrology, 900 Hospital of the Joint Logistics Team, Fuzhou, 350025 Fujian China; 6 Fujian Medical University, Fuzhou, 350025 Fujian China; 7 School of Life Science, Xiamen University, Xiamen, 361102 Fujian China

**Keywords:** Aptamers, NGAL protein, Acute kidney injury

## Abstract

**Background:**

Neutrophil gelatinase-associated lipocalin (NGAL) is a promising biomarker of early diagnosis and prediction for acute kidney injury (AKI). However, the current program for NGAL detection is not extensively applied in clinics due to the high expense of antibodies. Nucleic acid aptamers are single-strand DNAs or RNAs which could bind to targets with high specificity and affinity, and they have been widely used in the diagnosis and therapy for multiple diseases. It is valuable for us to develop a new method for NGAL detection using aptamers instead of antibodies to achieve increased efficiency and decreased cost.

**Methods:**

Nucleic acid aptamers against NGAL were obtained after SELEX process using magnetic beads, and an enzyme-linked aptamer analysis (ELAA), which can be widely used in clinical diagnosis at low cost, were successfully established. The feasibility of ELAA was further validated with urine samples harvested from 43 AKI patients and 30 healthy people.

**Results:**

Three candidate aptamers, including NA36, NA42 and NA53, were obtained after 8 rounds of SELEX process with magnetic beads and verified by quantitative polymerase chain reaction (qPCR), and the Kd value of each aptamer was 43.59, 66.55 and 32.52 nM, respectively. Moreover, the linear relationship was consistent at the range of 125–4000 ng/mL, and the detection limit of ELAA assay was 30.45 ng/mL. We also found that NGAL could be exclusively detected with NA53, and no cross-reaction between NA53 and human albumin or globulin occurred, the coefficient of variation (CV) between inner-plate and inter-plate was less than 15%, and the recovery rate was between 80 and 110%. Moreover, the sensitivity and specificity of ELAA assay in this study are 100% and 90%, respectively. Consistently, these results could also diagnose whether the occurrence of AKI in lots of patients, which has been demonstrated with the ELAA method we established after using NA53.

**Conclusions:**

Taken together, NA53, the best candidate aptamer targeting NGAL protein, can be applied in clinical testing.

**Electronic supplementary material:**

The online version of this article (10.1186/s12967-019-1955-7) contains supplementary material, which is available to authorized users.

## Background

Acute kidney injury (AKI) is a common clinical critical disease with high incidence accompanied by a sudden loss of renal function and urine output of < 0.5 mL/kg/h for more than 6 h [1]. The clinical symptoms of AKI include sustained increase in urea nitrogen and creatinine in serum and imbalance of saline. The incidence rate of AKI is 5–7% in patients and up to 30% in critical patients with 50% mortality [2]. Currently, serum creatinine is the most common biomarker used for AKI diagnosis [3], whereas it only increases when kidney function is irreversibly damaged. Therefore, biomarkers for early diagnosis of AKI should be explored to improve the cure and survival rate of AKI patients [1].

Neutrophil gelatinase-associated lipocalin (NGAL), also known as lipocalin 2, is a secreted protein possessing 178 amino acids in the form of monomers, homodimers and heterodimers [1], It is widely expressed in neutrophils, kidney, prostate, respiratory and digestive tract epithelial cells [4] and plays a vital role in natural immunity [5], anti-oxidation [6], tumorigenesis and tumour metastasis [7, 8]. A previous study showed that NGAL was markedly increased in the very early stages of AKI [1]. Low expression of NGAL was normally found in mature peripheral blood neutrophils, human tissues and organs. However, once surgery-related inflammation and renal tubular epithelial cell injury occurred, the concentration of NGAL rapidly increased in blood and urine, and it closely correlated with the AKI severity [9]; thus, it may be served as a powerful indicator in clinical application [10].

Previous methods established for NGAL protein detection have mainly been based on enzyme-linked immunosorbent assay (ELISA) with its antibody; however, this method cannot be popularised due to its high cost, and new methods and reagents should be developed for AKI diagnosis. Nucleic acid aptamers, a class of single-stranded DNA (ssDNA) or RNA [11], have special 3-dimensional structures, and they can be used as probes or recognition elements in disease diagnosis and therapy [12]. The process of their binding to targets resembles antigen–antibody reactions; thus, nucleic aptamers are considered as small molecules, which is better than antibodies since they are stable and hypoallergenic [11]. Moreover, they may be synthesised or amplified on a large scale and undergo various modifications at low cost.

In this study, we aimed to screen nucleic acid aptamers against NGAL with high specificity and high affinity using SELEX process with magnetic beads, thereby establishing a detection method based on ELAA to achieve convenient and accurate diagnosis in clinical settings.

## Materials and methods

### Chemicals, ssDNA and cell lines

NGAL protein (Cat No: 10222-H08H) and anti-lipocalin-2 antibody (Cat No: 10222-MM04) were obtained from Sino Biological (China). PCR Taq Mix (Cat No: AS111), qPCR Mix (Cat No: AQ131), Trans5α chemically competent cells (Cat No: CD201) and pEASY-T5 zero cloning kits (Cat No: CT501) were obtained from Transgen Biotech, China. A 20 bp DNA Ladder (Cat No: D521a) was obtained from the Takara Company. BioMag^®^ Plus Carboxyl Protein Coupling Kit (Cat No: BP611) was purchased from Bangs Laboratories. Bovine serum albumin (Cat No: A8020) and streptavidin/horseradish peroxidase (HRP) (Cat No: SE068) were obtained from Solarbio. 2× TBE-urea sample buffer (Cat No: C506046) and urea (Cat No: A600148) were purchased from the Sangon Company. Fluorochrome-conjugated secondary antibody (Cat No: A11005) was obtained from the Life Technology Company. DAPI-FLUOROMOUNT-G (Cat No: 0100-20) was acquired from the Southern Biotech company. All chemicals and reagents used were of analytical grade. The ssDNA in the library has a total length of 75 nt, which possesses a random 35 nt sequence in the middle of ssDNA and a fixed 20 nt sequence at both ends of ssDNA (Sangon Biotech Company). The ssDNA library dissolving in the ddW and the primers used for PCR are listed in Additional file 1: Table S1. HEK393T, A498 and cak-i 786-o and KYSE450 cells were obtained from the American Type Culture Collection (ATCC). Cells were cultured in RPMI Medium Modified (Cat No: SH30809, Hyclone, USA) supplemented with 10% fetal bovine serum (FBS; Gibco).

### Coating the magnetic beads

Fifty microliters of carboxyl magnetic beads (20 mg/mL) were washed four times with 500 μL of compound 2-(*N*-morpholino)ethanesulfonic acid (MSE) buffer. After the final wash, the beads were resuspended in 500 μL of MSE buffer, and 1.6 mg of 1-ethyl-3-(3-dimethylaminopropyl)carbodiimide (EDAC) was added to the resuspended beads. The flask was placed on a non-magnetic rotator for 30 min at room temperature. The supernatant was aspirated and discarded, followed by washing with 500 μL of MSE buffer 4 times. Fifty micrograms of NGAL was resuspended in 500 μL of MSE buffer, and 450 μL of NGAL solution (0.1 μg/μL) was added to the activated magnetic beads while shaking. Magnetic beads were harvested after shaking for 16-24 h, followed by resuspension in 500 μL of MSE buffer to repeat the steps described above. After adding 500 μL of quenching solution and shaking at room temperature for 30 min, magnetic beads were collected and added to 500 μL of wash buffer. After washing 4 times, the beads were resuspended with 200 μL of wash buffer. The coating efficiency was calculated with the formula: (Cpre-coating − Cpost-coating)/Cpre-coating × 100%. In addition, we prepared the bare beads using the same method except the replacement of NGAL solution with MSE buffer.

### SELEX assays

The magnetic beads were coated with NGAL following the manufacturer’s instructions. The negative screening method was performed as follows: 150 μL of 10 μM ssDNA library was soaked in boiling water for 5 min, then in ice-water for 3 min and centrifuged for 30 s at 12,000 rpm; 20 μL of bare magnetic beads was added and incubated for 30 min at room temperature. Positive screening was performed as follows: the supernatant derived from negative screening was harvested. Then, 30 μL of magnetic beads coated with NGAL were added and incubated at room temperature for 1 h. These beads were washed with 200 μL of binding buffer twice. Beads resuspended with 100 μL of ddw were added and heated in boiling water for 5 min, and the supernatant was transferred to new clean tubes. PCR amplification of the selected aptamer library used the following procedure: PCR reaction system (product after screen 4 μL, P3 4 μL, P4 4 μL, 2× PCR Taq mix 50 μL, ddw 38 μL), PCR reaction conditions (95 °C 5 min, 95 °C denaturation 30 s, 60 °C annealing 30 s, 72 °C extension 30 s, 20 cycles, 72 °C for another 5 min), and the product of PCR amplification was concentrated with butanol. The expected band was harvested using 10% 7 M urea denatured polyacrylamide gel electrophoresis (PAGE) gel and purified. The purified product was dissolved in 100 μL of binding buffer, and it was the sub-library for the next round of screening. The binding rate of NGAL in each round of SELEX was calculated using the formula: NGAL binding rate = NssDNA/[N − 1) ssDNA × M_NGAL_] × 100%. NssDNA and (N − 1) ssDNA represent the amount of eluted ssDNA and added ssDNA, respectively, and M_NGAL_ represents the amount of NGAL.

### qPCR verification assay

The enrichment library was used to perform T/A cloning following the instruction of the kit. Sequence alignment of nucleic acid aptamers was conducted using DNAMAN 8.0 software after sequencing.

qPCR was used to validate the aptamers according to the following procedure: T/A plasmid containing candidate aptamer was diluted to 100-fold, and 1 μL of diluted plasmid served as a template for PCR amplification. PCR reaction system: template 5 μL, P3 2 μL, P4 2 μL, 2× PCR Taq Mix 25 μL, ddw 16 μL; and the condition for PCR was the same as that described above. The PCR product was purified and dissolved in 200 μL of Tris–EDTA (TE) buffer. Fifty microliters of 20 nM aptamers were added to 1 μL of NGAL magnetic beads and bare magnetic beads, respectively. After incubation at room temperature for 1 h, these beads were put on the magnetic rack until the liquid is clear and then discarded the supernatant. The above steps were repeated 3 times after using 200 μL of binding buffer. One hundred microlitres of ddw was used to resuspend beads, and 1 μL of resuspended beads served as the template for qPCR (template 1 μL, P1 0.5 μL, P2 0.5 μL, 2× qPCR mix 7.5 μL, ddw 5.5 μL). The condition for qPCR reaction was performed as follows: 95 °C for 5 min (95 °C for 10 s, 60 °C for 10 s, 72 °C 10 s) for 20 cycles and 72 °C for 5 min. The ΔCt value represents the binding rate of aptamer to NGAL, and the criterion for selecting candidate aptamers based on the ΔCt value between the NGAL coated beads and bare beads once it is greater than 4 cycles.

### Comparison of the NGAL protein binding amount with the candidate aptamers

The candidate aptamers NA10, NA36, NA42, NA53, and NA21 were synthesised by the Sangon Company and diluted to 200 nM using binding buffer. Fifty microliters of 200 nM aptamers were added to 1 μL of NGAL magnetic beads and bare magnetic beads, respectively. After incubation for 1 h at room temperature, the beads were put on the magnetic rack until the liquid is clear and then the supernatant was discarded. Two hundred microlitres of binding buffer was added, and the procedure was repeated as described above 4 times. The magnetic beads were resuspended with 20 μL of ddw and diluted to 100-fold, and 1 μL of resuspended magnetic beads served as the template for qPCR to compare the binding amount of NGAL protein with these candidate aptamers.

### Specificity and affinity analysis of aptamers

Magnetic beads coated with normal human serum were produced according to the procedure afore mentioned, and the concentration of normal human serum was tenfold as high as that of NGAL. Fifty microlitres of 200 nM aptamer and 1 μL of pure magnetic beads were mixed and incubated for 1 h at room temperature, and these beads were put on the magnetic rack until the liquid is clear and then discarded the supernatant. Magnetic beads were resuspended in 20 μL ddw and diluted to 100-fold, and 1 μL resuspended magnetic beads served as a template for qPCR.

These candidate aptamers were diluted to a series of concentrations: 200 nM, 100 nM, 50 nM, 25 nM, 12.5 nM, 6.25 nM, 3.125 nM and 0 nM. Fifty microlitres of diluted aptamers possessing different concentrations as above were added to 1 μL of NGAL-coated magnetic beads and pure magnetic beads, respectively. After incubation for 1 h at room temperature, these beads were put on the magnetic rack until the liquid is clear, and then the supernatant was discarded. Magnetic beads were resuspended in 20 μL of ddw and diluted to 100-fold, and 1 μL of resuspended magnetic beads served as a template for qPCR.

### Establishment of ELAA method and its performance evaluation

Fifty microlitres of NGAL antibody (300 ng) was added to 96 well plates at 4 °C overnight, and washed the plates 3 times with buffer. After the addition of 350 μL of 3% BSA and incubation at 37 °C for 4 h, the plates were washed 3 times. Following the addition of NGAL and incubation for 1 h at room temperature, the plates were washed 3 times. One hundred microlitres of 200 nM biotinylated aptamer was added, and after incubation at room temperature for 1 h, the plates were washed 3 times. One hundred microlitres of 1/2,000 horseradish peroxidase-conjugated streptavidin (HRP-SA) were added, and the plates were washed 3 times. Subsequently, they were mixed with 100 μL of TMB and incubated at room temperature for 20 min, and the reaction was terminated using 50 μL of stop solution. The optical density (OD) values at the wavelength of 450 nm was measured by a microplate reader.

The specificity of the ELAA method was evaluated as follows: the OD value of 4 μg/mL NGAL protein, 400 μg/mL albumin, 400 μg/mL globulin and binding buffer were simultaneously tested using ELAA method. The standard curve of the ELAA method was evaluated as follows: NGAL protein was diluted to different concentrations, such as 4000 ng/mL, 2000 ng/mL, 1000 ng/mL, 500 ng/mL, 250 ng/mL, 125 ng/mL, 62.5 ng/mL, 31.25 ng/mL, 15.625 ng/mL and 0 ng/mL, and the OD value of these samples were tested with the ELAA method.

The sensitivity of the ELAA method was evaluated as follows: standard curves of NGAL were analysed based on the OD value when the concentration of NGAL was 0 ng/mL, 15.625 ng/mL, 31.25 ng/mL and 62.5 ng/mL. The OD value of twenty blank samples were tested, and then the minimum detection limit was calculated based on the sum of the mean OD value of blank samples with twofold SD value. The precision of the ELAA method was evaluated as follows: the OD value of NGAL solution at 250 ng/mL, 1000 ng/mL and 2000 ng/mL concentration were tested, the inner-plate and inter-plate precision were analysed, respectively.

The accuracy of the ELAA method was evaluated as follows: the urine of 5 healthy people were mixed and served as the basic sample. Then, NGAL protein was added to this mixture to preparing recycle sample 1 and recycle sample 2, and the concentration of NGAL in these 2 mixtures reached 250 ng/mL and 1000 ng/mL, respectively. Following this, the OD value of the mixture, recycle sample 1 and recycle sample 2 were tested using the ELAA method, and the recycle rate was calculated using the formula: recycle concentration/input concentration × 100%.

### Immunohistochemistry

Cells were fixed with paraformaldehyde (PFA) for 15 min at room temperature and rinsed 3 times in phosphate-buffered saline (PBS) for 5 min, and the cells were treated with 2% TritonX-100 in PBS for 5 min, then rinsed with PBS and blocked with blocking buffer for 60 min. After incubation with FAM-NA53 for 60 min, they were rinsed with 1% TritonX-100 in PBS in a Thermostatic shaker at 60 °C 3 times. These cells were incubated with diluted primary antibody overnight and protected from light. Then, the cells, which were pre-rinsed with PBS, were incubated with diluted flurochrome-conjugated secondary antibody for 2 h at room temperature and covered with DAPI-FLUOROMOUNT-G, and the pictures of cellular morphology were obtained with a Nikon fluorescence microscope (Ts2-FL).

### Clinical sample harvest and clinical tests

All clinical samples in this experiment were obtained from the 900 Hospital of the Joint Logistics Team during 2017–2019 with the authorisation of patients and complied with the ethical regulations. According to the criteria of Kidney Disease: Improving Global Outcomes (KDIGO) for AKI diagnosis, patients who have any following characteristics could be diagnosed as AKI patients: (1) the level of serum creatinine (Scr) increased more than 0.3 mg/dL (or more than 50%) within 48 h; (2) the level of Scr increased more than 1.5-fold of baseline value within 7 days; (3) the volume of urine is less than 0.5 mL/kg/h in 6 h. Thirty-four urine samples were harvested from 43 patients with AKI. Among these clinical samples, 35 urine samples were males and 8 urine samples were females, and the ages of these patients ranged from 15 to 78 years old, with a mean age of 47 years. Moreover, another 30 urine samples derived from healthy people. There were 20 males and 10 females between 20 and 72 years old, with a mean age of 37. Detailed information and corresponding creatinine levels of these samples are listed in Additional file 1: Table S2. The fresh urine samples of patients with AKI and healthy control were harvested and stored at − 80 °C. The urine of 73 samples were harvested, respectively, and the concentration of NGAL protein in these samples was tested using the ELAA method and the results were analyzed with the Mann–Whitney U test. The optimal cut off value used for distinguishing patients with AKI and healthy control and the sensitivity and specificity of ELAA method were obtained using ROC curve. More importantly, 4 urine samples, which were derived from both the acute and recovery stages of AKI, were used for monitoring the changes of NGAL protein and creatinine with ELAA method and pircric acid method, respectively.

### Statistical analysis

Differences between groups were compared using analysis of variance for repeated measures. A two-tailed Student’s t-test was used to analyse the age and creatinine, and the comparison of NGAL protein level was analysed with Mann–Whitney U test, standard curve and ROC curve were analysed using excel and SPSS20.0 software, respectively. Other data analyses were performed using GraphPad PRISM. v5.0 software (San Diego, CA, USA), and p value < 0.05 was considered significant.

## Results

### Aptamers targeting NGAL are successfully obtained using SELEX process

**Fig. 1:**
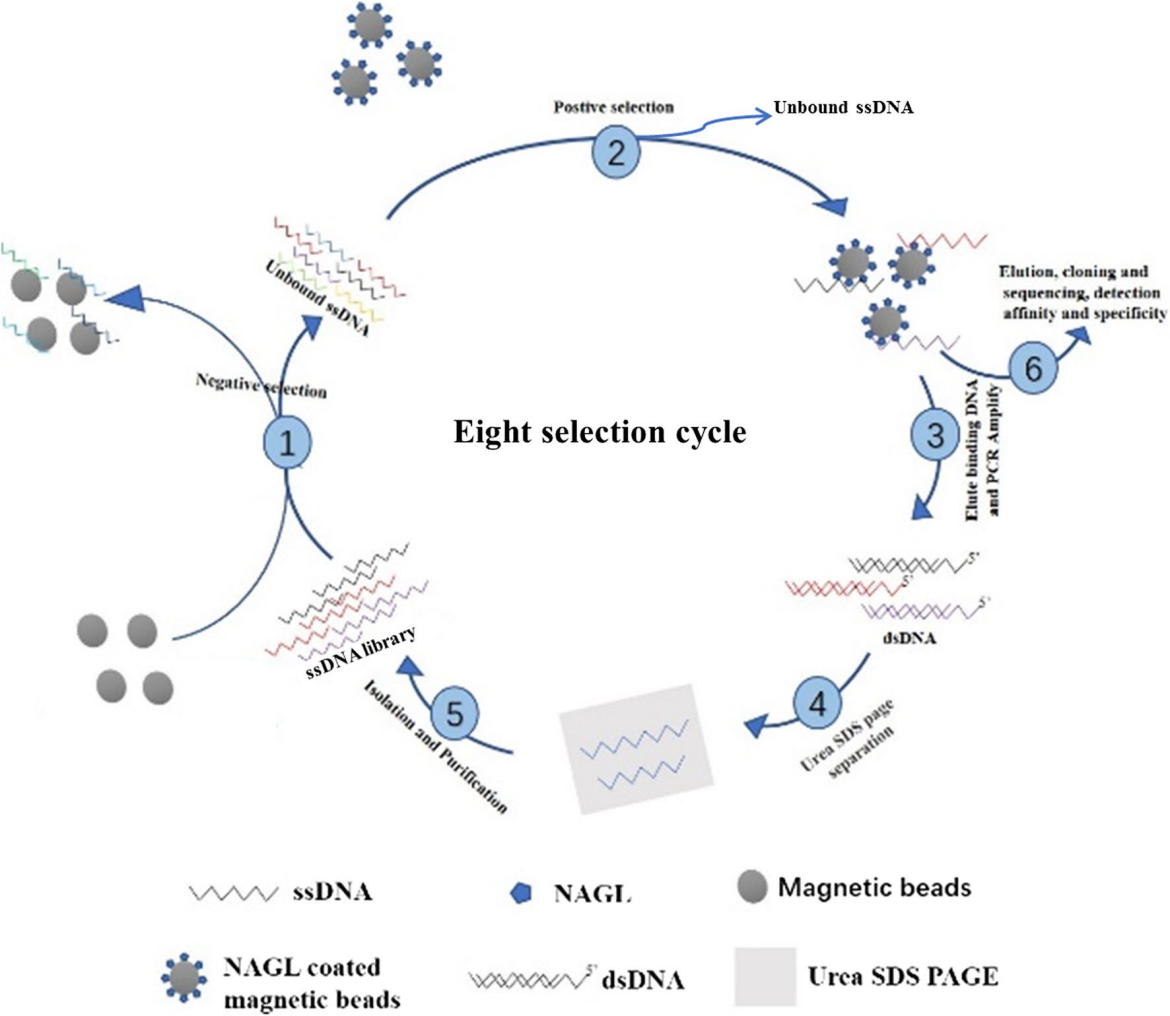
The schematic flow for the magnetic SELEX process. Multiple steps were involved in the SELEX process, including ssDNA pool synthesis, negative selection, positive selection, elution, cloning, sequencing and SDS-PAGE separation

After coating with NGAL protein, the coating efficiency was determined to be up to 91.4% (Additional file 2: Figure S1), namely, 0.21 μg NGAL protein was coated with 1 μL of magnetic beads, and the concentration we used was adjusted to 0.20 μg NGAL/μL of magnetic beads for the process of screening. The outline of the SELEX procedure is shown in Fig. 1. In this study, 8 rounds of SELEX screening with magnetic beads were used to obtain NGAL-specific nucleic acid aptamers. During the screening process, the pressure of screening gradually increased through the modulation of multiple parameters, including the amount of NGAL, aptamer library input, incubation time, the times of washing and shaking (Additional file 1: Table S3). Additionally, before the SELEX process, we tested the optimal conditions for PCR amplification when using the aptamer library as the template. We chose 60 °C to be the best annealing temperature, and 1.0 μL of 10 μM primer served as the best input in the PCR reaction (Additional file 2: Figure S2A). Moreover, we monitored the result of negative control at every round of the SELEX process, and no template pollution was confirmed throughout all processes (Additional file 2: Figure S2B).

**Fig. 2:**
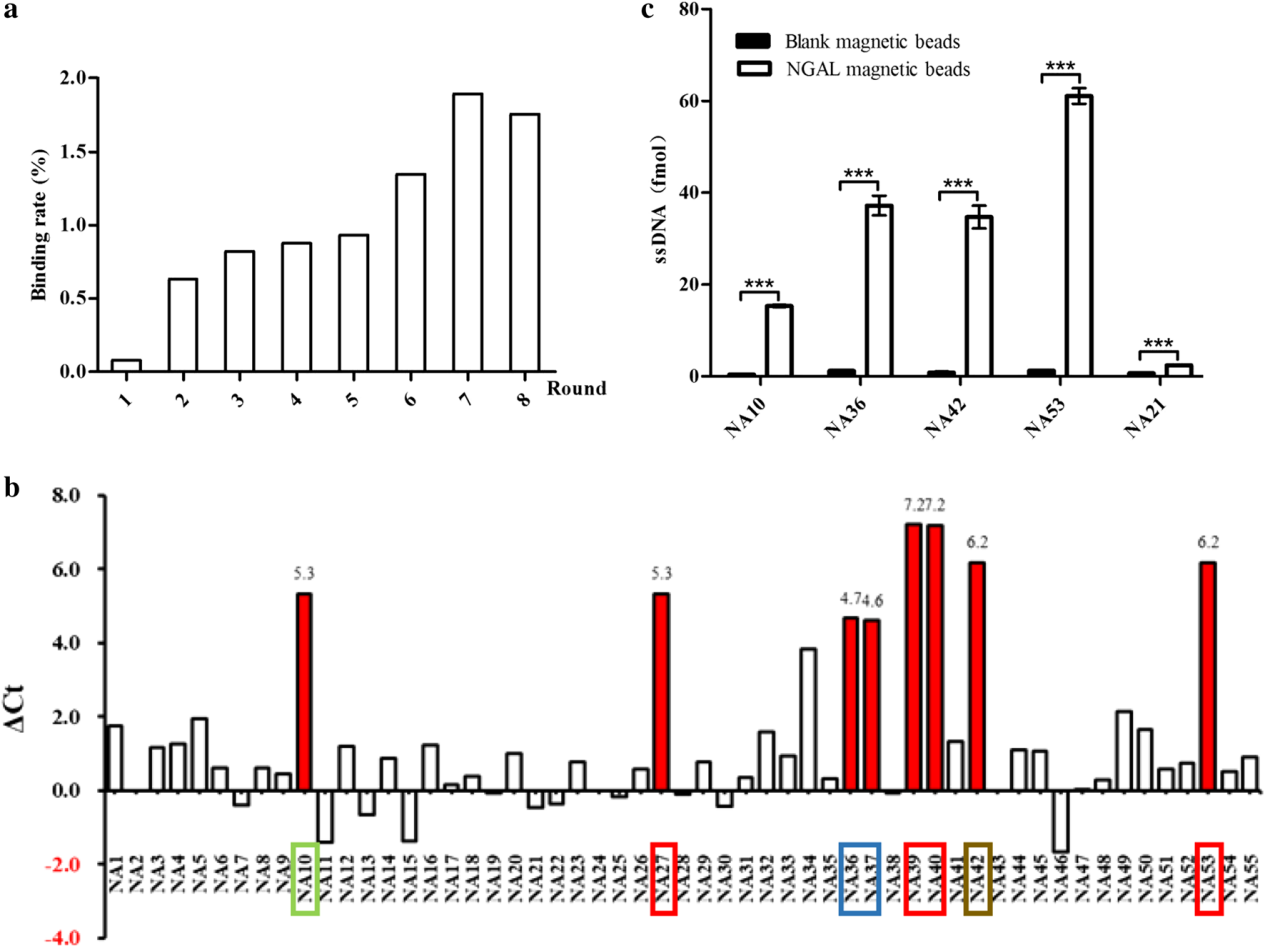
Specific aptamers obtained after multiple rounds of the SELEX process. **a** The binding rate of 1 μg of NGAL protein during every SELEX round, and the binding rate of NGAL protein increased from the initial 0.079% at round 1 to 1.89% at round 7. **b** The binding status between 53 aptamers and NGAL protein were primarily validated using the quantitative method, and ΔCt greater than 4 served as the selection standard. **c** The binding amounts of candidate aptamers, including NA10, NA36, NA42, NA53 and NA21 to NGAL-coated magnetic beads are much higher than those of bare magnetic beads using the qPCR method (n = 3, for each group, *p < 0.05, **p < 0.01, ***p < 0.001 vs control, and the error bars indicate the mean ± standard deviation SD)

To monitor the process of SELEX screening, the binding rate was used to reflect the enrichment of the aptamer pool. As shown in Fig. 2a, the binding rate of NGAL protein increased from the 0.079 to 1.89% with screening rounds proceeded. However, we noticed that the binding rate did not increase or even decreased after the 7th round of screening, which may be caused by the strengthened pressure for screening and the limitation of the binding of aptamers to targets. Therefore, we used the solution eluted from the 8th round of screening for T/A cloning and sequencing.

Fifty-five monoclonal colonies were chosen for sequencing, and they were nominated to be NA1–NA55, respectively. As shown in Additional file 1: Table S4, NA2 and NA43 are blank vectors, and the homology of residual aptamers was analysed using DNAMAN 8.0 (Additional file 2: Figure S2C). Moreover, NA39, NA40 and NA53 are completely identical, and NA27 has one different base when comparing with NA39 aptamers, while NA36 and NA37 have the same sequences as NA20 and NA21. Moreover, the homology of the other 45 aptamers is not high, suggesting that the aptamer library was enriched, and the screening process was successful. Thus, we validated and compared these 53 aptamers using the qPCR method. As shown in Fig. 2b, 53 plasmids produced by T/A cloning, which contain aptamers, were used for PCR amplification. After incubation with bare magnetic beads or NGAL-coated beads, the washed beads served as templates. We found that the ΔCt values of most of these 53 aptamers were more than 0, indicating that the aptamers obtained after the 8th round of the SELEX screening process were enriched. Intriguingly, the ΔCt value of aptamers, which comprise NA10, NA27, NA36, NA37, NA39, NA40, NA42 and NA53, was more than 4, and 4 of them, NA10, NA36, NA42, and NA53, were chosen as candidate aptamers and further study, including homology analysis using qPCR.

**Fig. 3:**
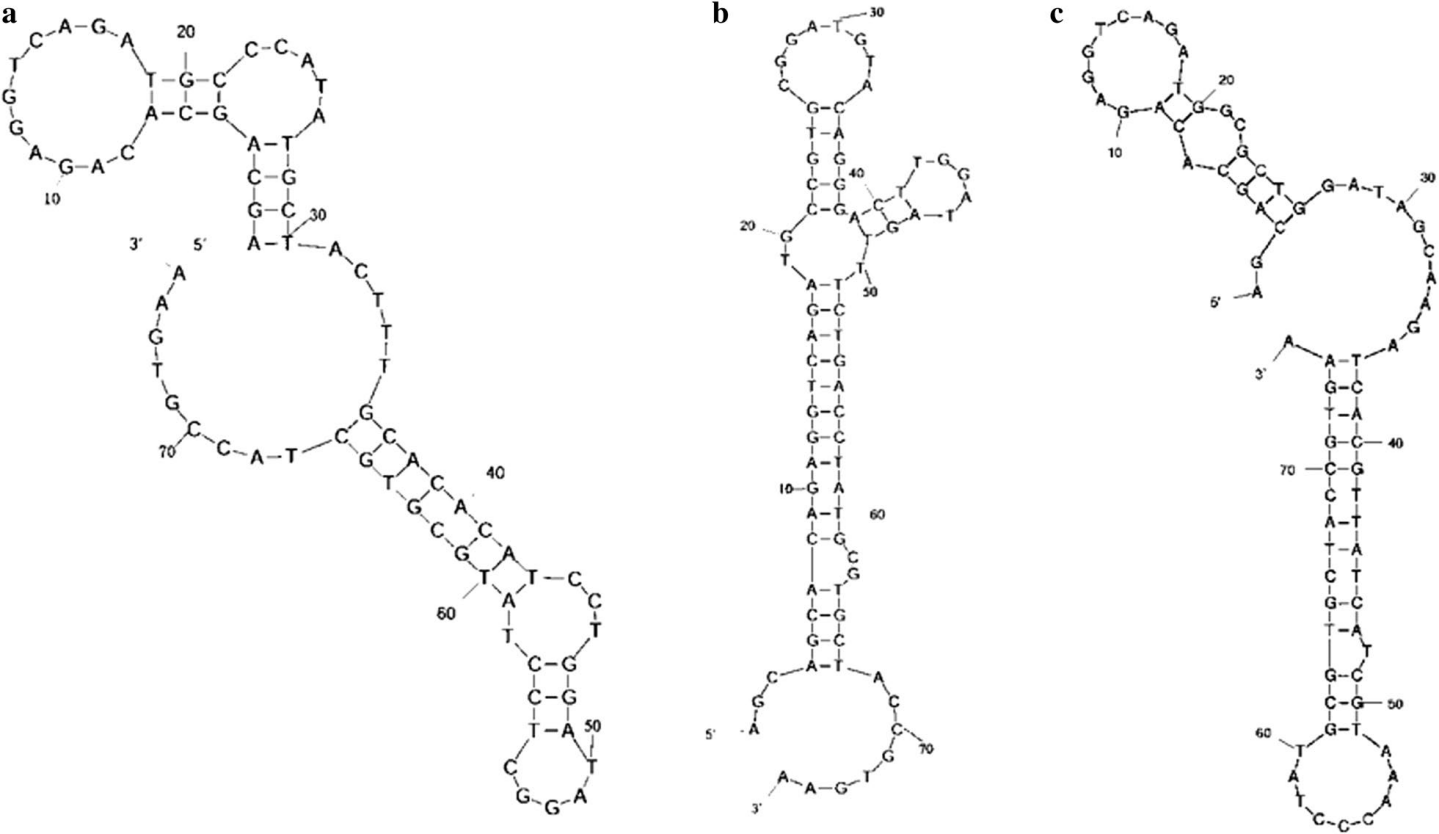
The predicted secondary structure of NA36, NA42 and NA53 aptamers. **a** The predicted secondary structure of NA36 aptamers. **b** The predicted secondary structure of NA42 aptamers. **c** The predicted secondary structure of NA53 aptamers

The sequencing result of NA21 was verified by the qPCR method when comparing with other candidate aptamers NA10, NA36, NA42, and NA53, and we found that NA10 and NA21 were not suitable for further studies due to their low capability of binding to NGAL-coated beads (Fig. 2c). The primary sequences of **NA36**, **NA42** and **NA53** were AGCAGCACAGAGGTCAGATG**CCCAT ATGCTACTTTGCACACATCCTGGATAGGCT**CCTATGCGTGCTACCGTGAA for NA36, AG CAGCACAGAGGTCAGATG**CCGTGCGGATGTACAGGGACTTGGATAGTTTCTGA**CCTATGCGTGCTACCGTGAA for NA42 and AGCAGCACAGAGGTCAGATG**GCGCTGGATAGCAA GATCACGTTATCATCGTAAAC**CCTATGCGTGCTACCGTGAA for NA53, respectively. Moreover, the secondary structure of aptamers NA36, NA42 and NA53 were analysed using online tools (http://unafold.rna.albany.edu/?q=mfold/DNA-Folding-Form). The condition for the structure prediction is 25 °C, 0.1 M Na^+^ and 0.001 M Mg^2+^, and the result of prediction is consistent with our results (Fig. 3).

### Specificity and affinity analysis of the candidate aptamers

**Fig. 4:**
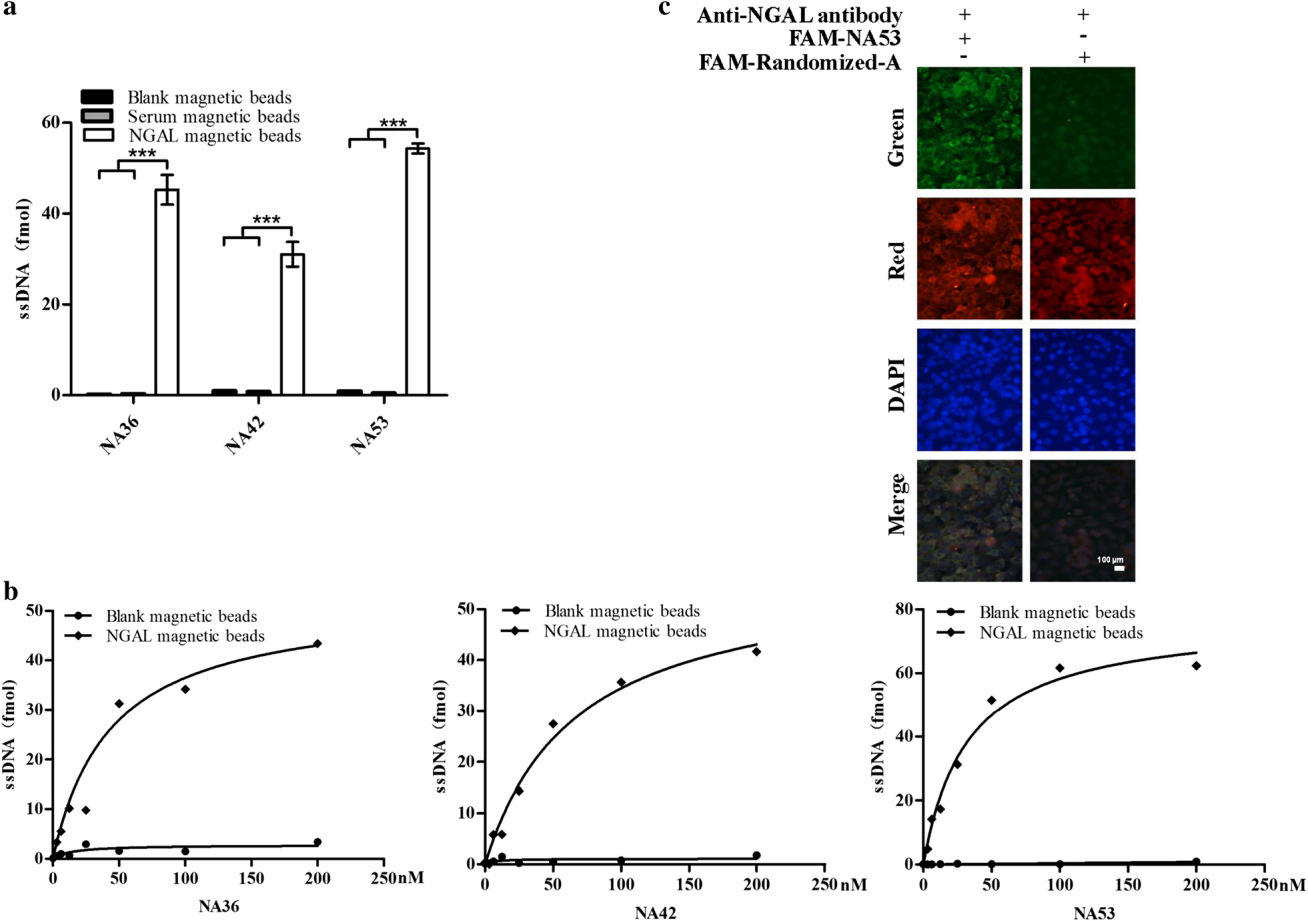
The analysis of the specificity and affinity between the candidate aptamers and NGAL protein. **a** The specificity assay of the binding status between aptamers and bare magnetic beads, NGAL-coated beads, and normal human serum-coated beads using the qPCR method. **b** The Kd value of candidate aptamers was analysed after serial dilution, and NA53 aptamers possess the strongest binding capability on NGAL proteins. **c** NGAL protein and NA53 are colocalised in oesophageal squamous carcinoma cells KYSE450 using immunohistochemistry. Herein the green channel represents the interactions between aptamer library or NA53 and NGAL protein, the red channel represents the interactions between NGAL antibody and NGAL protein, and the blue channel represents the occurrence of nuclear after staining with DAPI, and the merged channel represents the overlap of green channel, red channel and blue channel

NA36, NA42 and NA53 aptamers were incubated with blank magnetic beads, NGAL-coated beads and normal human serum-coated beads, respectively. The amount of aptamers, which were bound to these 3 types of magnetic beads, was measured and monitored by qPCR. As shown in Fig. 4a, there were many more aptamers bound to the NGAL-coated beads than the other 2 types of beads, suggesting that these 3 aptamers could specifically bind to NGAL protein.

NA36, NA42 and NA53 aptamers were diluted to be several different concentrations, such as 200 nM, 100 nM, 50 nM, 25 nM, 12.5 nM, 6.25 nM, 3.125 nM and 0 nM, and then they were incubated with bare beads and NGAL-coated beads, respectively. Subsequently, qPCR was used to determine the amount of aptamers binding to beads, and the Kd value was calculated using GraphPad Prism 5 (Fig. 4b), and we found that Kd values of NA36, NA42 and NA53 aptamers was 43.59, 66.55 and 32.52 nM, respectively (Figs. 2c, 4a), suggesting that NA53 aptamer has the strongest capability on target binding to NGAL among these three aptamers.

Additionally, NA53 aptamers was validated to be the optimal aptamers among these 3 aptamers, which may potentially replace the antibody of NGAL, and thus it may also be used for the detection of NGAL protein and imaging. Moreover, our results further showed that NA53 could colocalise with NGAL proteins in KYSE450 cells, one type of oesophageal squamous carcinoma cells, using immunohistochemistry; however, when we used the random libraries as controls, the signal of colocalisation of NGAL protein and aptamers could not be detected, suggesting that NA53 could specifically bind to NGAL protein (Fig. 4c).

### Establishment of ELAA method using NA53

**Fig. 5:**
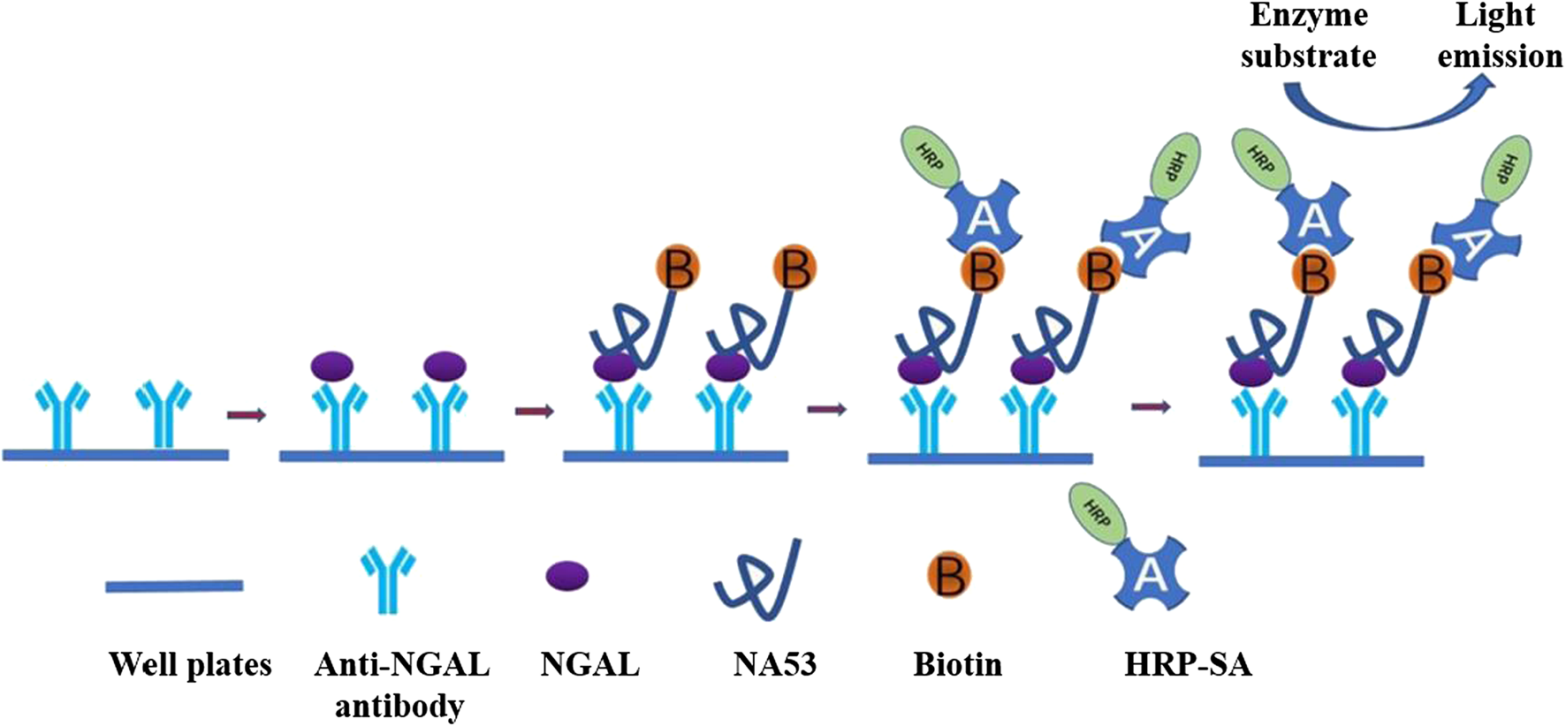
Schematic illustration of the ELAA method established in this study. The ELAA detection method was established using the strategy of sandwich structure of antibody-NGAL-NA53 aptamers

To establish a feasible method for detecting NGAL protein using the NA53 aptamers, we designed an ELAA detection method based on the strategy of sandwich model, and the structure of sandwich model consists of NGAL antibody, NGAL protein and NA53 aptamers (Fig. 5), and the model could be destroyed once any component is deficient, resulting in an OD value less than 0.05 (Additional file 2: Figure S3A). However, the OD value of the group, a reaction system that possesses the components of NGAL antibodies, NGAL protein and NA53 aptamers, was 1.8, and the OD value of the group, which possesses NGAL antibodies, NGAL protein and NGAL antibodies, was 1.6.

In the ELAA assay, the amount of NGAL antibody coated in a 96-well plate is critical for its establishment. In a high-adsorption 96-well plate, 0 ng, 25 ng, 50 ng, 100 ng, 300 ng, 500 ng and 700 ng of NGAL antibodies were coated respectively, and we found that the OD value tended to be stable and no longer increased once the amount of NGAL antibodies used was more than 300 ng, which represents an optimal amount of antibody for coating (Additional file 2: Figure S3B).

**Fig. 6:**
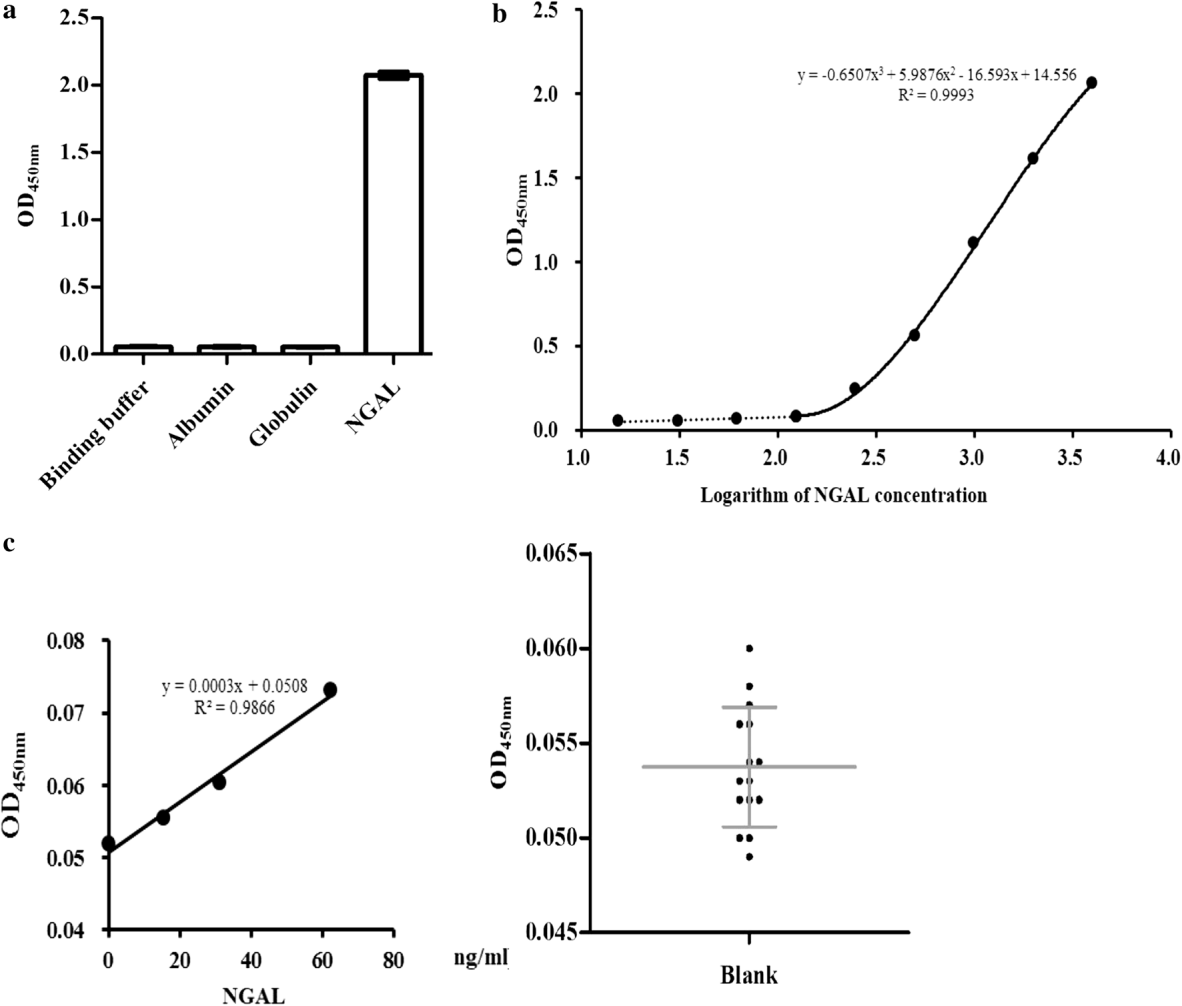
The performance verification of the ELAA method established with NA53 aptamers. **a** The ELAA method with NA53 aptamers can detect NGAL without cross-reaction with the albumin and globulin after specificity analysis. **b** The establishment of standard curve and the detection range of the ELAA method. **c** The sensitivity analysis of the ELAA method with NA53 aptamers

NGAL protein in serum and urine has been used for the diagnosis and prediction of AKI, while NGAL protein in urine is more useful for its high specificity. Therefore, the ELAA detection method we established was mainly used to detect the level of NGAL in urine. Our specificity analysis showed that there were no cross-reactions between the common albumin and globulin in urine. Moreover, the OD value of binding buffer, albumin (400 μg/mL) and globulin (400 μg/mL) were approximately 0.05, while the OD value of NGAL (4 μg/mL) was 2.05 (Fig. 6a). These results revealed that the ELAA method we established with NA53 aptamers can exclusively detect the NGAL protein, and no cross-reaction with albumin and globulin occurred.

The NGAL stock solution was diluted to serial concentrations between 0 and 4000 ng/mL for ELAA assay. The fitting cubic equation was y = − 0.6507x^3^ + 5.9876x^2^ − 16.593x + 14.556, R^2^ = 0.9993 (Fig. 6b), x represents NGAL concentration and y represents the corresponding OD value in the formula, respectively, and the range of detection was 125–4000 ng/mL. When the concentration of NGAL was less than 62.5 ng/mL, the formula for calculation was y = 0.0003x + 0.0508, R^2^ = 0.9866, the OD value of all 15 blank controls was approximately 0.05 and the detection limit was estimated to be 30.45 ng/mL (Fig. 6c).

We evaluated the precision of the ELAA method in this study. The results of 3 concentrations of the same plate were listed in Additional file 1: Table S5, and the CV value was less than 10%. The results obtained from 2 different plates are shown in Additional file 1: Table S6, and the CV value was less than 15%. In addition, we evaluated the accuracy of the ELAA method described above, and we found that the recycle rate of these 2 different concentrations of NGAL were 80% and 110% (Additional file 1: Table S7).

### Application of ELAA with NA53 in clinical trials

**Fig. 7:**
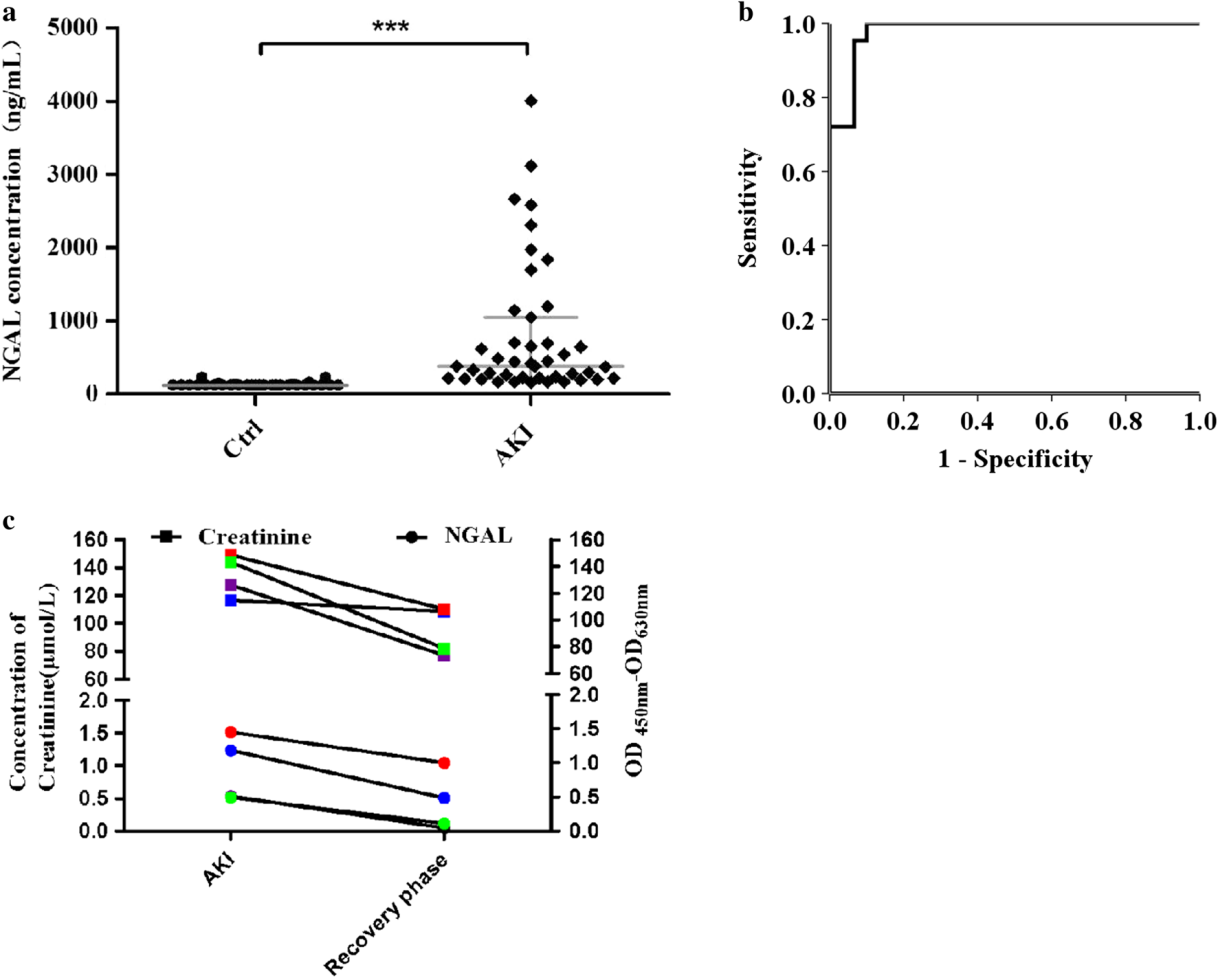
The ELAA method established with NA53 aptamers can distinguish AKI patients in clinical samples. **a** NGAL protein level in AKI patients is significantly higher than its level in healthy samples after application of the ELAA method with NA53 aptamers. **b** NGAL protein diagnostic value and ROC curve analysis. **c** The level of NGAL protein in urine of 4 AKI patients decreased from the acute phase to recovery phase with the ELAA method, which is consistent with the change of creatinine (each colour in the figure represents one patient with AKI)

In this study, we tested the feasibility of applying NA53 in clinical trials. We firstly tested the feasibility of ELAA method with NA53 in a series of urine samples, which derived from 30 healthy samples and 43 patients with AKI in the 900 Hospital of the Joint Logistics Team. As shown in Fig. 7a, we found that the concentrations of NGAL protein in 24 samples of 30 healthy people is lower than 125 ng/mL, which is not in the examination range. However, the concentration of NGAL in all 43 patients with AKI is located in the examination range, and their levels of NGAL protein are significantly higher than the healthy controls (P < 0.001); thus the result indicates that the method we established with NA53 aptamer can be used to distinguish the patients and healthy people.

In addition, according to the results of ROC curve (Fig. 7b), when the minimum concentration of NGAL, namely the cut off value, is 157.04 ng/mL, the maximum value of Yodden index is 0.90, and the corresponding AUC is 0.98, the sensitivity and specificity of the ELAA method we established were 100% and 90%, respectively, thus our result here is consistent with previous study [13]. Futhermore, the level of NGAL protein and creatinine were further monitored with the urine of 4 AKI patients during the acute and recovery phases (Fig. 7c), and we found that the level of NGAL protein in the later phase was significantly lower than its level in the acute phase, which is also consistent with the tendency of creatinine, suggesting that the level of NGAL protein could be used to monitor the status of recovery stage of patients with AKI.

## Discussion

AKI is one of the most common diseases, and the classical biomarker for this disease is the serum creatinine level. Many biomarkers have been proposed for its early diagnosis, including kidney injury molecule-1 [14], interleukin 18 [15, 16], cystatin C [17], liver-type fatty acid-binding protein [18], *N*-acetyl-β-d-glucosaminidase and NGAL protein [19]. Among these biomarkers, NGAL protein in the urine was mainly derived from the kidney during AKI, but not from neutrophils, hepatocytes or respiratory and intestinal tract epithelial cells. Moreover, in the urine of animal models of gentamicin-induced acute kidney disease, early increase of the serum creatinine level was not obvious. However, the level of NGAL protein was significantly increased, suggesting that NGAL protein may be a sensitive indicator for the early diagnosis of AKI [20, 21].

To improve the prevention, diagnosis, treatment and prognosis prediction of AKI, the exploration of novel and ideal biomarkers, which are especially used for the early diagnosis is very important. An ideal biomarker should be organ specificity, high sensitivity and a good sign of reflecting the severity of diseases accompanied by the increased level of biomarkers. In addition, simplicity, noninvasiveness and low cost are also required. Although several biomarkers described above have been explored and reported for the early diagnosis of AKI, other biomarkers have lower specificity and sensitivity for AKI diagnosis than NGAL protein, and their time used for diagnosis is also longer than NGAL protein [1, 9, 14–17, 22, 23]. The level of NGAL protein rapidly increased in the blood and urine during the very early stage of AKI, and it can distinguish prerenal and renal AKI [24], and the level of NGAL protein in urine could be an independent predictor which can reflect the status of recovery from AKI [25]. Taken together, NGAL protein meets the criteria of ideal biomarkers for AKI diagnosis. Increased level of NGAL protein is also found when infection with bacteria and virus or malignant tumours occur [7, 8, 26, 27], thus the status of patients and differential measures for treatment should be comprehensively analysed.

Previously, only antibodies have been used to detect their targets in scientific research and clinical settings. Currently, people found that aptamers can also bind to their targets via Van der Waals forces, hydrogen bonds, electrostatic forces and hydrophobic forces with high specificity and affinity. The binding process is also similar to the formation of antibody-antigen complexes; therefore, aptamers are also called “chemical antibodies”, and the dissociation constant (Kd value) of aptamers usually varies from pM to nM [28]. Compared with antibodies, advantages of aptamers are long half-life, nontoxic, and they can also be produced with targets which do not have immunogenicity. Moreover, they can also be easily modified and combined with other materials to achieve better effect based on our requirements; therefore, they have great potential in multiple applications including detection, diagnosis, imaging, drug delivery and the development of anti-cancer drugs [29, 30].

In this study, we used traditional method to perform SELEX screen against NGAL protein with magnetic beads, meanwhile, the result of SELEX process, the affinity and specificity of primary aptamers were further validated with qPCR method. Moreover, we found that aptamer NA53 can directly bind to the membrane of KYSE450, a stable cell line of esophageal squamous cell carcinoma, suggesting that the aptamer NA53 can bind to NGAL protein with high specificity and affinity, obviously, our study is different to previous study published by Lee group [13]. Moreover, the detection method based on aptamer contains two domains, one is the targeting domain, namely aptamer, and another is the signaling domain including radionuclide or fluorescent [31]. According to the signals and strategies used for detection, ELAA method can be mainly developed to multiple methods including colorimetric method, chemiluminescence method, electrochemical method, fluorescence method. The most common detection method based on aptamers always contains a sandwich structure, which is formed with target, two specific aptamers or one specific aptamer and the antibody of target. The ELAA method, which was established using aptamer NA53 to replace NGAL antibody, could be widely used for diagnosing AKI patients after the combination of the clinical significance of NGAL protein and the advantages of nucleic acid aptamers.

Currently, the main difficulty of applying aptamers in clinics is the environment and condition in research is not always same to clinics, and the effect of aptamers will be impaired or disappeared since the structure of aptamers will change under different conditions, such as the change of pH value or ion concentration in the environment. Although the NGAL protein in blood and urine samples can both be used for disease diagnosis and prediction, however the NGAL protein in urine is more suitable to be the early marker for AKI diagnosis and prediction than in serum. As we now know, the NGAL protein in urine is a specific response marker after renal injury, and the NGAL protein in serum is easily interfered by inflammation, infection, tumor and other factors, thus the specificity of NGAL protein in serum is not as specific as it in urine [32]. Therefore, our study used urine samples in clinical test since they have many advantages, for example, the composition of urine are relatively simple, the process of urine harvest is convenient and no wound. Additionally, the binding of aptamer NA53 to NGAL protein in urine was also validated after performance verification. More importantly, no cross-reactions occurred between aptamer NA53 and other main components in urine including albumin and globulin after test with ELAA method, thus the ELAA method in our study is worth popularizing for NGAL protein test.

Our study showed that the sensitivity and speficity of NGAL protein are 100% and 90% after test with 73 urine samples using ELAA method and aptamer NA53, respectively. However, the sensitivity and speficity of other traditional markers are inferior to NGAL protein in our study, such as 61% and 61% with serum creatinine [33], 82.2% and 76.4% with serum cystatin C [34], 70.8% and 90.9% with pNGAL [35], and 77.1% and 73.3% with urinary NGAL protein [36], respectively. Our results revealed that the NGAL protein in urine has higher sensitivity and specificity as AKI diagnosis with ELAA method, which was established with aptamer NA53. Compared with clinical tests with antibodies, the production period of aptamer is shorter, and its chemical synthesis and modification are easier, and the cost of clinical test with aptamer is lower. Of note is ELAA detection method has many advantages, such as high sensitivity, high specificity and lower cost, and it is easily popularised. However, the interactions between aptamers and targets in ELAA method are also easily affected by environmental factors. Although the ELAA method we established can detect the NGAL protein in urine samples, however the time for its application is longer, and the repeatability of its application in our study may be slightly worse when compared with other ELAA detection methods based on aptamers, such as chemiluminescence method and electrochemical method, therefore it should be further optimized to achieve extensive use in clinical tests in the future.

## Conclusions

In this study, we screened and obtained the aptamers against NGAL protein after multiple rounds of SELEX process. As an important indicator, NGAL protein can rapidly enter the serum and urine of AKI patients; moreover, it has higher sensitivity and specificity [9]. Therefore, it is meaningful for us to obtain the specific reagent and to establish effective method for NGAL protein detection. We found that NA53 is the optimal aptamer among the 3 candidates after performance comparison and verification. Application of NA53 in the ELAA method, which was established with the replacement of the second antibody in ELISA with NA53, has good performance for the detection of NGAL protein, the limit of detection (LOD) is 30.45 ng/mL and the linear relationship is constant at the range of 125–4000 ng/mL, and the sensitivity and specificity are 100% and 90%, respectively, therefore it could be potentially exploited for clinical tests. Notably, detection of NGAL protein in clinics may be in high demand, and using NA53 with ELAA method, but not the second antibody with the ELISA method, will save the cost in clinics.

## Additional files

**Additional file 1.** Additional tables.

**Additional file 2.** Additional figures.

## Data Availability

The datasets supporting the conclusions of this article are included within the article, and the materials will be available once request.

## Abbreviations

AKI: acute kidney injury
AMD: age-related macular degeneration
CV: coefficient of variation
EDAC: 1-ethyl-3-(3-dimethylaminopropyl)carbodiimide
ELAA: enzyme-linked aptamer analysis
ELISA: enzyme-linked immunosorbent assay
HRP-SA: horseradish peroxidase-conjugated streptavidin
LOD: limit of detection
MSE: 2-(*N*-morpholino)ethanesulfonic acid
NGAL: neutrophil gelatinase-associated lipocalin
OD: optical density
PAGE: polyacrylamide gel electrophoresis
PBS: phosphate-buffered saline
PFA: paraformaldehyde
qPCR: quantitative polymerase chain reaction
Scr: serum creatinine
ssDNA: single-stranded DNA
SD: standard deviation
TE: tris-EDTA
