# Supplementary material for: Development of a novel ssDNA aptamer targeting neutrophil gelatinase-associated lipocalin and its application in clinical trials
Source: J Transl Med. 2019 Jun 18;17:204. doi: 10.1186/s12967-019-1955-7 (PMC6582607; doi:10.1186/s12967-019-1955-7)
Supplement: Supplementary file 1 — Additional file 1. Additional tables. [file 12967_2019_1955_MOESM1_ESM.docx]

Table S1. The sequence of ssDNA library and primers

|  | Sequence (5’ → 3’) |
| --- | --- |
| ssDNA library | 5’-AGCAGCACAGAGGTCAGATG-N_35_-CCTATGCGTGCTACCGTGAA-3’ |
| Forward primer | 5’-AGCAGCACAGAGGTCAGATG-3’ |
| Reverse primer | 5’- TTCACGGTAGCACGCATAGG-3’ |
| FAM-P3 | 5’- FAM-AGCAGCACAGAGGTCAGATG-3’ |
| PolyA-P4 | 5’-AAAAAAAAAAAAAAAAAAAA-link-TTCACGGTAGCACGCATAGG-3’ |

Table S2. The information of clinical samples

|  | Normal | AKI | p |
| --- | --- | --- | --- |
| Cases | 30 | 43 | - |
| Gender（M/F） | 20/10 | 8/35 | - |
| Age | 36.63±12.21 | 47.07±17.47 | 0.04 |
| Concentration of creatinine(umol/L) | 76.54±14.73 | 201.59±153.41 | 0.001 |

Table S3. The parameters used for optimization in the 8 rounds of SELEX screening pressure

| SELEX Round  （150μL） | NGAL（μg）  （μL） | ssDNA  （pmol） | Incubation time（min） | Wash times（times） | Rotating speed（r/min） |
| --- | --- | --- | --- | --- | --- |
|  |  |  |  |  |  |
| 1 | 6（30） | 1400 | 60 | 2 | 400 |
| 2 | 6（30） | 243.20 | 60 | 2 | 400 |
| 3 | 5（25） | 230.07 | 50 | 3 | 400 |
| 4 | 5（25） | 205.96 | 50 | 3 | 600 |
| 5 | 4（20） | 149.79 | 40 | 4 | 600 |
| 6 | 4（20） | 119.66 | 40 | 4 | 600 |
| 7 | 3（15） | 98.57 | 30 | 5 | 800 |
| 8 | 2（10） | 73.60 | 30 | 5 | 800 |

Table S4. The sequence of obtained aptamers after T/A cloning and sequencing

| Aptamers | Sequence alignments of aptamers |
| --- | --- |
| NA7 | AGCAGCACAG AGGTCAGATG **ACCGCATGAG GATCTCCATC TATCGACCCT GAGGA**CCTAT GCGTGCTACC GTGAA |
| NA8 | AGCAGCACAG AGGTCAGATG **ATTCCCTTAG GGTGTCCATG CGTGGTTCTC ATTAC**CCTAT GCGTGCTACC GTGAA |
| NA9 | AGCAGCACAG AGGTCAGATG **ACACTGGACA CTAACGTTAC CTATTAGACG TCCTG**CCTAT GCGTGCTACC GTGAA |
| NA10 | AGCAGCACAG AGGTCAGATG **TGCTGGAATC AACACGATCG CATCCCGGAT GGGCT**CCTAT GCGTGCTACC GTGAA |
| NA11 | AGCAGCACAG AGGTCAGATG **GCTGCAGTTT TGACGGCGAA GATCTTCCTA GGGTT**CCTAT GCGTGCTACC GTGAA |
| NA12 | AGCAGCACAG AGGTCAGATG **ATGAAGGATG CCGACCGAGT TCTTTGCTTC CGATT**CCTAT GCGTGCTACC GTGAA |
| NA13 | AGCAGCACAG AGGTCAGATG **TTGGTTTTCT GGGAAGATCG TGCGAATATT ATGCA**CCTAT GCGTGCTACC GTGAA |
| NA14 | AGCAGCACAG AGGTCAGATG **TATCGGCAAG TGTAGTATAC TAGCACGTCC CTCTT**CCTAT GCGTGCTACC GTGAA |
| NA15 | AGCAGCACAG AGGTCAGATG **TTCCCCTCAG CATTCCGTCT CCGTTCTATA GCTCA**CCTAT GCGTGCTACC GTGAA |
| NA16 | AGCAGCACAG AGGTCAGATG **GTCCTCCGGG CCACAAAGGC TCTTTAATTT TCCTG**CCTAT GCGTGCTACC GTGAA |
| NA17 | AGCAGCACAG AGGTCAGATG **GCGCGTGTAT CCTTCATCAA CGGACCTTGG GCGTA**CCTAT GCGTGCTACC GTGAA |
| NA18 | AGCAGCACAG AGGTCAGATG **GGGAGCGTAC GGATGTGTAG ATTGGTCCCG CTTCG**CCTAT GCGTGCTACC GTGAA |
| NA19 | AGCAGCACAG AGGTCAGATG **TCCATCGATT TGGCGCCAAC CCTATACTAA** CCTATGCGTG CTACCGTGAA |
| NA20 | AGCAGCACAG AGGTCAGATG **CAACGGGTAT AACCAGATGA TTTGGCGCCA CGTAA**CCTAT GCGTGCTACC GTGAA |
| NA21 | AGCAGCACAG AGGTCAGATG **CAACGGGTAT AACCAGATGA TTTGGCGCCA CGTAA**CCTAT GCGTGCTACC GTGAA |
| NA22 | AGCAGCACAG AGGTCAGATG **CGGACTGGAC TGATCCCGGC TGTTTCATGT CCCCCC**CCTA TGCGTGCTAC CGTGAA |
| NA23 | AGCAGCACAG AGGTCAGATG **AAGGCCGGGG TAGGAAGAAT GCTTATTGTA TTCCG**CCTAT GCGTGCTACC GTGAA |
| NA24 | AGCAGCACAG AGGTCAGATG **TTTTCTCGTA GATCGTCTCG GCCTTCGTGT CCTTT**CCTAT GCGTGCTACC GTGAA |
| NA25 | AGCAGCACAG AGGTCAGATG **CGTCGGTCAT GAATCAGTAC GTCCTCTATG TGTT**CCTATG CGTGCTACCG TGAA |
| NA26 | AGCAGCACAG AGGTCAGATG **GGACTTTTTT ACGCCTTTCA TTTCGAGTGA GCGTG**CCTAT GCGTGCTACC GTGAA |
| NA27 | AGCAGCACAG AGGTCAGATG **GCGCTGGATA GCAAGATCAC GTTATCGTCG TAAAC**CCTAT GCGTGCTACC GTGAA |
| NA28 | AGCAGCACAG AGGTCAGATG **TCGTTCGTGT GTGGGCCCTT CCGGTTTTCA CGATC**CCTAT GCGTGCTACC GTGAA |
| NA29 | AGCAGCACAG AGGTCAGATG **TCCAGGCGCA TGGGGCAACC AACTGTAATG AGACA**CCTAT GCGTGCTACC GTGAA |
| NA30 | AGCAGCACAG AGGTCAGATG **GGTATTACTC CCGCATCACC AGTCTTACGT TCAGT**CCTAT GCGTGCTACC GTGAA |
| NA31 | AGCAGCACAG AGGTCAGATG **TTTAACGCGC GAGTAGGGCT ATTCTGTGTT CCAGT**CCTAT GCGTGCTACC GTGAA |
| NA32 | AGCAGCACAG AGGTCAGATG **GCGAGAATTT CTGGCGCTGG CCCGCGATAC GTTTC**CCTAT GCGTGCTACC GTGAA |
| NA33 | AGCAGCACAG AGGTCAGATG **ATATGTGCCT AGAGGTGGGG TATGATTACA AACGT**CCTAT GCGTGCTACC GTGAA |
| NA34 | AGCAGCACAG AGGTCAGATG **ACTTTGGATC CCAAGAACAA ATCTCCATCC TAAAC**CCTAT GCGTGCTACC GTGAA |
| NA35 | AGCAGCACAG AGGTCAGATG **ACAACCCGGC ATCCAGCATC TACCTGCAAG CGGAC**CCTAT GCGTGCTACC GTGAA |
| NA36 | AGCAGCACAG AGGTCAGATG **CCCATATGCT ACTTTGCACA CATCCTGGAT AGGCT**CCTAT GCGTGCTACC GTGAA |
| NA37 | AGCAGCACAG AGGTCAGATG **CCCATATGCT ACTTTGCACA CATCCTGGAT AGGCT**CCTAT GCGTGCTACC GTGAA |
| NA38 | AGCAGCACAG AGGTCAGATG **ACTGAGATAA TCTTGAGTTA TGTCTAGATG TACGG**CCTAT GCGTGCTACC GTGAA |
| NA39 | AGCAGCACAG AGGTCAGATG **GCGCTGGATA GCAAGATCAC GTTATCATCG TAAAC**CCTAT GCGTGCTACC GTGAA |
| NA40 | AGCAGCACAG AGGTCAGATG **GCGCTGGATA GCAAGATCAC GTTATCATCG TAAAC**CCTAT GCGTGCTACC GTGAA |
| NA41 | AGCAGCACAG AGGTCAGATG **ACTGAATTCA GGCTGGATTA TCCTGTGCGT TTG**CCTATGC GTGCTACCGT GAA |
| NA42 | AGCAGCACAG AGGTCAGATG **CCGTGCGGAT GTACAGGGAC TTGGATAGTT TCTGA**CCTAT GCGTGCTACC GTGAA |
| NA44 | AGCAGCACAG AGGTCAGATG **GGCCGTCTCT CTACACTTGT CCATGTCCTC CGCCT**CCTAT GCGTGCTACC GTGAA |
| NA45 | AGCAGCACAG AGGTCAGATG **TAGCGTCGAG GAATGCGACT TTGAATTCAG ACCGT**CCTAT GCGTGCTACC GTGAA |
| NA46 | AGCAGCACAG AGGTCAGATG **GGCCTATTCC TTGGGGACTT CCGTTGAGCC ATAAC**CCTAT GCGTGCTACC GTGAA |
| NA47 | AGCAGCACAG AGGTCAGATG **CGGGATCTAT CCTCCGCGCA TGCCCTTTAG GCTTT**CCTAT GCGTGCTACC GTGAA |
| NA48 | AGCAGCACAG AGGTCAGATG **GGGGAGACGG CCACTACCGC TATTTATCTT GATAC**CCTAT GCGTGCTACC GTGAA |
| NA49 | AGCAGCACAG AGGTCAGATG **GAGATTGGGG CGATTTGTGG AGCGATTGGT TTGCC**CCTAT GCGTGCTACC GTGAA |
| NA50 | AGCAGCACAG AGGTCAGATG **GTTATGCCCC TGCACTGGGT TACAAGCGCC TGTAT**CCTAT GCGTGCTACC GTGAA |
| NA51 | AGCAGCACAG AGGTCAGATG **TCCCGGAGTA CTTCTCGCTC GTCCTTTTCC GTACT**CCTAT GCGTGCTACC GTGAA |
| NA52 | AGCAGCACAG AGGTCAGATG **CCGAGGCGTT CCTCCGACGT TTGTCCCGGG TCTGT**CCTAT GCGTGCTACC GTGAA |
| NA53 | AGCAGCACAG AGGTCAGATG **GCGCTGGATA GCAAGATCAC GTTATCATCG TAAAC**CCTAT GCGTGCTACC GTGAA |
| NA54 | AGCAGCACAG AGGTCAGATG **ATGCTTCCTG TTTTAATGCG CCCTATGTTC GAAGA**CCTAT GCGTGCTACC GTGAA |
| NA55 | AGCAGCACAG AGGTCAGATG **ATCGTCATGG TTTCTAGACG ATGGCTTTAG TTGGT**CCTAT GCGTGCTACC GTGAA |

Table S5. Inner-plate precision of ELAA

| Sample | 1 | 2 | 3 |
| --- | --- | --- | --- |
| Repeat times | 3 | 3 | 3 |
| Average | 269.23 | 816.63 | 1798.97 |
| SD | 17.29 | 11.17 | 155.79 |
| Coefficient of variation（%） | 6.42 | 1.37 | 8.66 |

Table S6. Inter-plate precision of ELAA

| Sample | 1 | 2 | 3 |
| --- | --- | --- | --- |
| Repeat times | 3×2 | 3×2 | 3×2 |
| Average | 254.13 | 958.87 | 1975.95 |
| SD | 16.82 | 118.53 | 101.59 |
| Coefficient of variation（%） | 6.62 | 12.36 | 5.14 |

Table S7. The result of recylce experiment

| Number | Basic Sample | Recyle sample 1 | Recyle sample 2 |
| --- | --- | --- | --- |
| Concerntration of NGAL Input（ng/mL） | 0 | 250 | 1000 |
| Concerntration of NGAL tested（ng/mL） | 160.20 | 432.67 | 1007.38 |
| Recycle Concerntration of NGAL（ng/mL） | — | 272.47 | 847.18 |
| Recycle Rate（%） | — | 108.99 | 84.72 |
