# Supplementary material for: Development of a novel ssDNA aptamer targeting neutrophil gelatinase-associated lipocalin and its application in clinical trials
Source: J Transl Med. 2019 Jun 18;17:204. doi: 10.1186/s12967-019-1955-7 (PMC6582607; doi:10.1186/s12967-019-1955-7)

**Figure S1. The result of coating for magnetic beads with NGAL protein.**

A. No band of NGAL protein occurs after coating.

B. The efficiency of coating with NGAL protein reaches 91.4%.


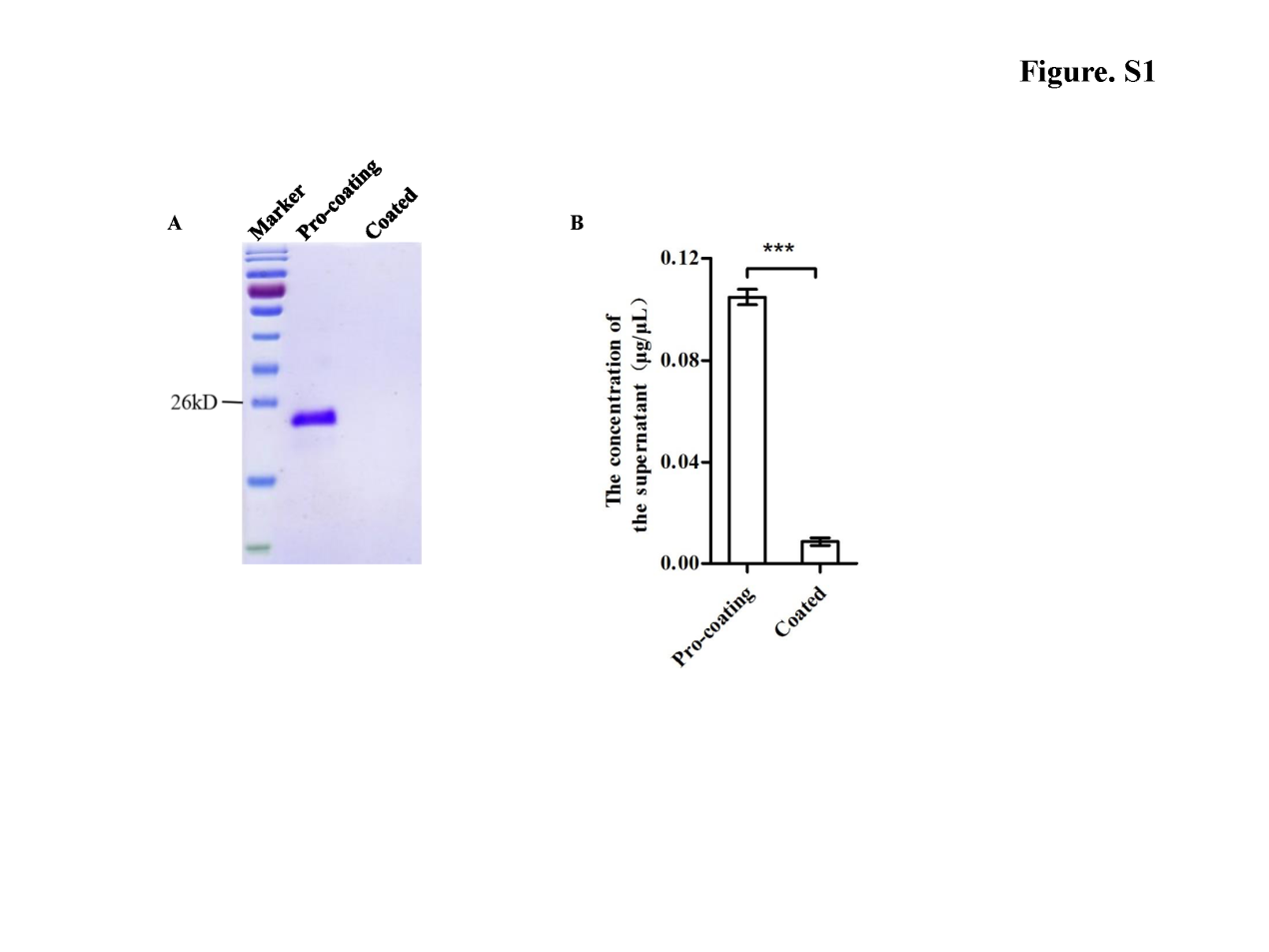


**Figure S2. PCR optimization and sequencing analysis result for aptamers SELEX process.**

A. The result of PCR condition optimization.

B. The agarose gel of negative control and PCR product after eight rounds of SELEX process.

C. The result of homology analysis for fifty three aptamers using DNAMAN software.


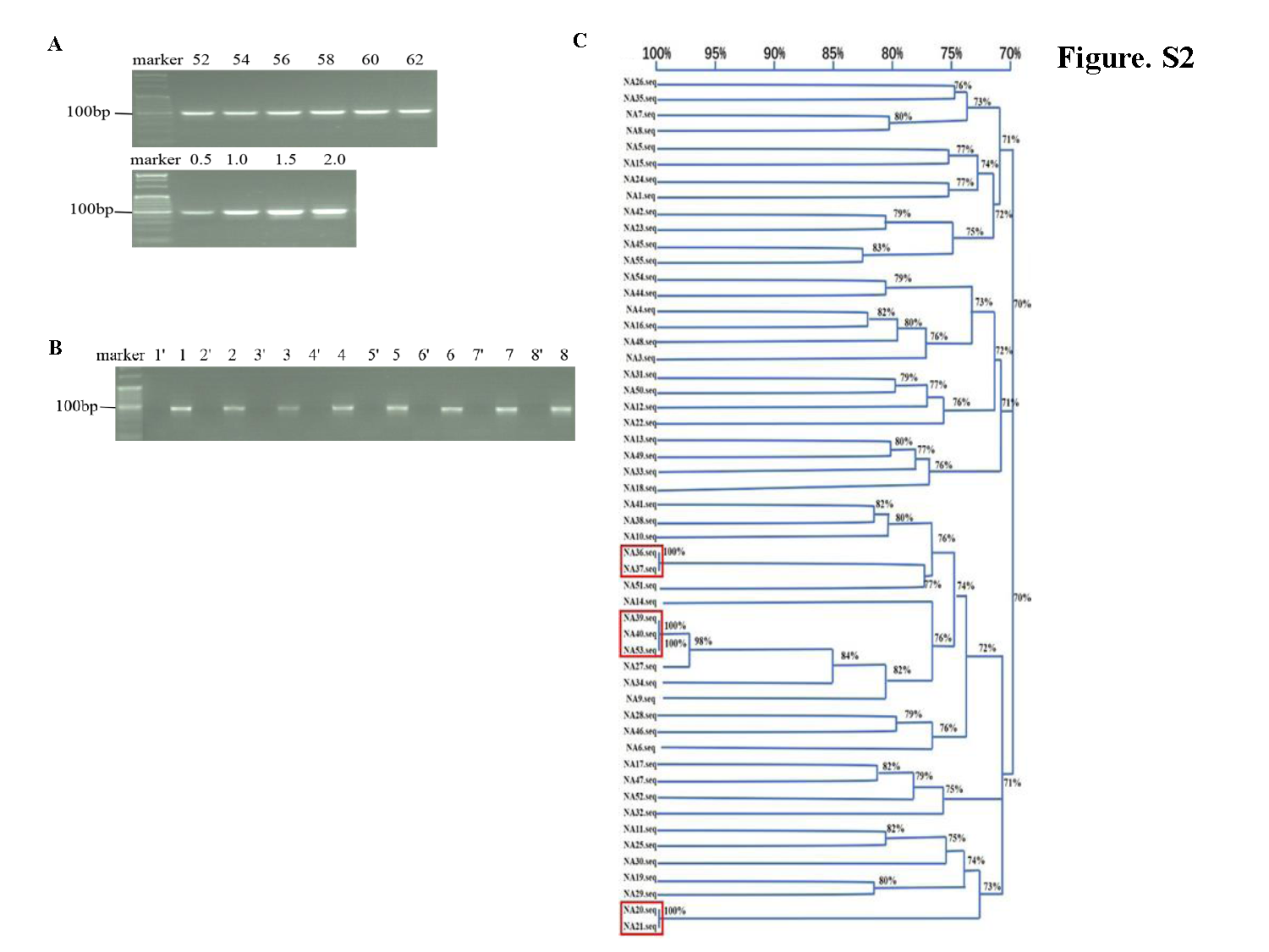


**Figure S3. The optimization for the condition of ELAA method**.

A. The result of feasible analysis for ELAA method with NA53 aptamers..

B. In a high-adsorption 96-well plate, 0, 25, 50, 100, 300, 500, and 700 ng of NGAL antibody were coated in every well, respectively. Once the amount of the coated NGAL antibody was greater than 300 ng, the OD value tended to be stable.


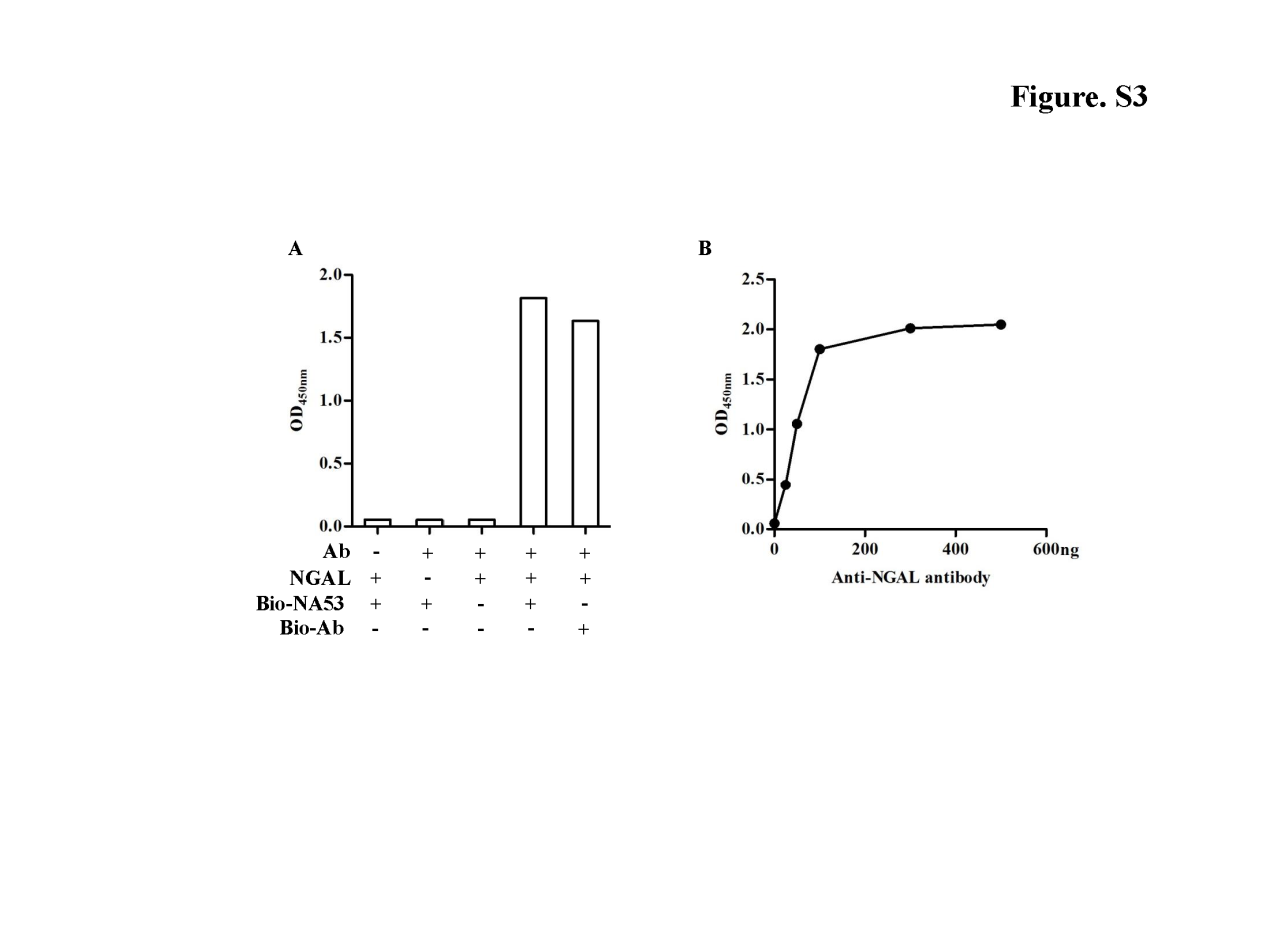

Supplement: Supplementary file 2 — Additional file 2. Additional figures. [file 12967_2019_1955_MOESM2_ESM.docx]
